# Migration and Fisheries of North East Atlantic Mackerel (*Scomber scombrus*) in Autumn and Winter

**DOI:** 10.1371/journal.pone.0051541

**Published:** 2012-12-10

**Authors:** Teunis Jansen, Andrew Campbell, Ciarán Kelly, Hjálmar Hátún, Mark R. Payne

**Affiliations:** 1 DTU AQUA - National Institute of Aquatic Resources, Technical University of Denmark, Charlottenlund, Denmark; 2 Fisheries Ecosystems Advisory Services, Marine Institute, Galway, Ireland; 3 Faroe Marine Research Institute, Tórshavn, Faroe Islands; 4 ETHZ Swiss Federal Institute of Technology, Zurich, Switzerland; University of Hamburg, Germany

## Abstract

It has been suggested that observed spatial variation in mackerel fisheries, extending over several hundreds of kilometers, is reflective of climate-driven changes in mackerel migration patterns. Previous studies have been unable to clearly demonstrate this link. In this paper we demonstrate correlation between temperature and mackerel migration/distribution as proxied by mackerel catch data from both scientific bottom trawl surveys and commercial fisheries. We show that mackerel aggregate and migrate distances of up to 500 km along the continental shelf edge from mid-November to early March. The path of this migration coincides with the location of the relatively warm shelf edge current and, as a consequence of this affinity, mackerel are guided towards the main spawning area in the south. Using a simulated time series of temperature of the shelf edge current we show that variations in the timing of the migration are significantly correlated to temperature fluctuations within the current. The proposed proxies for mackerel distribution were found to be significantly correlated. However, the correlations were weak and only significant during periods without substantial legislative or technical developments. Substantial caution should therefore be exercised when using such data as proxies for mackerel distribution. Our results include a new temperature record for the shelf edge current obtained by embedding the available hydrographic observations within a statistical model needed to understand the migration through large parts of the life of adult mackerel and for the management of this major international fishery.

## Introduction

Changes in global climate and the aspiration for sustainable fisheries management have highlighted the requirement for improved understanding of the effects of the marine climate on the behaviour of important fish species [1]. Mackerel (*Scomber scombrus*) is an abundant migratory pelagic fish in the north-east Atlantic, where it plays an important ecological role by feeding on zooplankton and on the pelagic larval and juvenile stages of a number of commercially important fish stocks [2], [3]. Furthermore, mackerel is itself targeted by whales, fish and a large pelagic fishing fleet with annual landings of between 500 000 and 1 000 000 tonnes [2], [4]. The largest mackerel fishery targets and follows mackerel aggregations throughout autumn and winter. Marked historical changes in the timing and spatial distribution of this fishery have been observed, but remain unexplained [4]–[7]. The fishing fleet is composed of modern pelagic trawlers and seiners that use sonar to locate schools of adult mackerel and are highly mobile, regularly steaming hundreds of kilometres from port. As a result of this adaptive behaviour, it is feasible that the observed changes in the timing and spatial distribution of commercial landings are representative of the spatiotemporal dynamics of the mackerel population.

It has been hypothesized that temperature is an important modulator of the autumn/winter spawning migration. An acoustic and oceanographic survey in December 1995 demonstrated a relationship between the location of mackerel in the Northern North Sea prior to the onset of migration and the local temperature field [8]. It has also been noted that mackerel behaviour appeared to be related to temperature while the mackerel stayed to the north and west of the Shetland [9], [10]. If the distribution of the fishery reflects the distribution of the mackerel and the mackerel distribution is related to the water temperature, then we would expect the temperature field to be reflected in the spatiotemporal distribution of the fishery. However, previous studies have not revealed any simple correlation between these variables [5]–[7].

Using fisheries independent data from scientific bottom trawl surveys and commercial landings statistics we investigate the mackerel migration from October to March and test

- whether data from commercial fisheries and scientific bottom trawl surveys can form the basis for useful proxies of the distribution of adult mackerel
- whether changes in the temperature of the shelf edge current are related to the significant temporal and spatial variation observed in these proxies

We consider our results in the light of other factors that influence the fishing fleet behaviour such as fisheries development, legislation and distance to home port. Finally, we discuss our findings within a larger oceanographic context of circulation patterns and global warming, review possibilities for hindcasts and forecasts, and implications for fisheries management.

## Materials and Methods

### Fisheries Data

**Figure 1:**
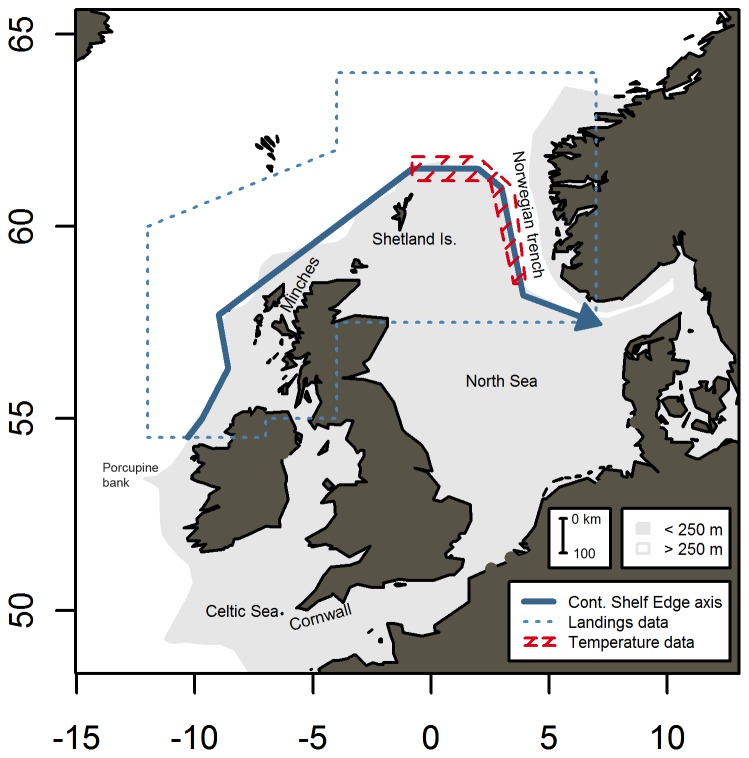
Map of study area and place names referred in the text. Continental shelf marked in grey (bottom depth <250 m). Blue polygon indicates the study area. Blue bold arrow shows the Continental shelf edge axis. Red shaded area marks the area of temperature profiles.

Quarterly landings in the autumn-winter fishery were used as reported to the International Council for Exploration of the Sea (ICES). Due to the fact that the autumn-winter fishery overlaps two calendar years, first quarter landings were treated as being a ‘5^th^’ quarter of the previous year. Thus, Q4 landings are those reported in October–December and Q5 corresponds to January–March of the following year. The study area encompasses the northern limit of the reported catches and includes the majority of the total reported catch (83% in Q4 and 56% in Q5) (Figure 1).

Commercial landings data were reported to ICES as quarterly totals per ICES statistical rectangle (1° latitude by 0.5° longitude). The position and time of the catch was assumed to be at the center of the reported rectangle and midway through the quarter. The landings consisted primarily (>95%) of adult fish [4].

To investigate the spatial variations in the behavior of the fleet the reported landings were projected onto a curvilinear ‘Continental Shelf Edge’ (CSE) axis in the style of [11], from 54.5 N 10.5 W in the south, and following the 200 m isobath, passing north of the Shetland Islands before turning south and following the Norwegian Trench into the North Sea (Figure 1). The total length of the CSE axis is approximately 1700 km. Each reported landing was projected onto the CSE axis by selecting the closest of 1000 equally spaced positions along the CSE axis. Distances were calculated based on great circle (WGS84 ellipsoid) distances. Both the position projected onto CSE axis and the distance of the reported landing from the axis were calculated and stored for further analysis.

The quarterly CSE axis distributions were then represented by a single metric for further comparison with temperature. Two alternative metrics were explored;

- the center of gravity of landings (CoG)
- the position of 50% cumulative landings (Po50%CL)

CoG was calculated by year and quarter as the weighted average of distances. The weighting factor was the mass (in kg) of each projected landing record. Po50%CL was calculated as the position along the CSE where the cumulative landings represented 50% of the total landings by year and quarter.

A literature survey and an interview with the skipper of a vessel that fished throughout the study period were carried out in order to identify periods where changes in the behavior of the commercial fishery were driven by factors other than mackerel behavior.

### Bottom Trawl Survey Data

Data from international bottom trawl surveys (IBTS) carried out in quarter 1 (January–March) between 1985 and 2011 on the shelf out to 500 m were downloaded from the ICES repository (http://datras.ices.dk). The study area was limited to the area described for the commercial landings. Relatively few mackerel were caught outside the study area, *e.g.* in Kattegat/Skagerrak [12] and over 90% were from surveys in March. Further south, in the Bay of Biscay, mackerel arrive at the spawning grounds around the time of this survey [13]: the present dataset therefore covers the northern part of the NEA mackerel population. Catch per Unit Effort (CPUE) of adult mackerel was calculated as catch in numbers per trawl hour, where adult mackerel were defined as being longer than 27 cm (most mackerel first spawn at the age of 2 (58%) and the mean length at age 2 in Q1 west of Scotland is 27 cm [4]). For ease of comparison with the commercial landings dataset, first quarter surveys were treated as being a ‘5^th^’ quarter of the previous year. Hauls were projected onto the CSE axis as described for commercial landings and the CoG and Po50%CL of CPUEs calculated.

### Temperature Data and Modelling

**Figure 2:**
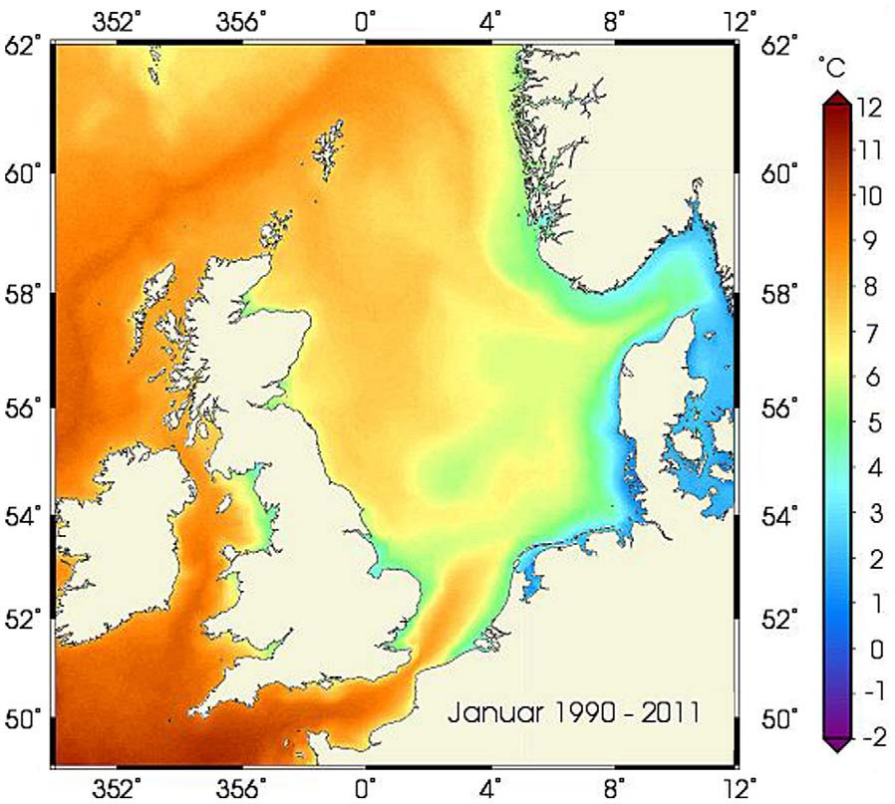
Map of average sea surface temperature in January 1990–2011 showing the relatively warm high-saline eastern Atlantic water flowing north-eastwards on and along the continental shelf edge, flanked by cooler water masses. Temperature measurement measured by satellite and mapped with permission from Bundesamt für Seeschifffahrt und Hydrographie, Germany (www.bsh.de).

In the present study, we investigate links between water temperature and mackerel distribution that could support the hypothesis of a temperature-driven migration. The continental shelf edge current which flows along the shelf edge to the northwest of Scotland, north and then east of the Shetland Islands, along the western edge of the Norwegian trench and into the northern North Sea, is warmer than both the surrounding coastal waters and the oceanic waters off the shelf during winter (Figure 2) [8], [9]. It is the temperature of this water mass that is of interest in this study. Unfortunately, relevant observations are not available for the entire study period. A relevant temperature record was therefore obtained by embedding the available hydrographic observations within a statistical model. The modelled area is shown in Figure 1 and was selected because it is the coldest area of the warm core of the current (Figure 2) and therefore the area where cold avoidance by mackerel would be most pronounced. Also, there are a significant number of observations available for this area.

It is within this core of relatively warm water in the northern North Sea that acoustic surveys found mackerel to aggregate in 50–220 m depth in early winter [8]–[10], [14]. Due to the fact that water is cooled throughout the winter, both downstream (along) and away from the CSE, temperature was modeled with year, day of year, distance parallel (CSE) and perpendicular (dCSE) to the CSE axis as explanatory variables i.e:
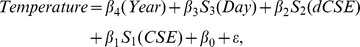
where *CSE* is the distance along the CSE axis from the start of the axis (in the south) to the projected sample position, *dCSE* is the distance from the sample site to the projected position, *day* is the number of days elapsed in the year, from 1^st^ of February (day 32) to 31^st^ of January (Day 386). *Year* is the year of the observation and, *S*() is the penalized cubic regression spline smoothing function implemented in the “mgcv”-R-package as cardinal spline [15]. *Day*, *CSE* and *dCSE* were thus modeled as smoothed predictor variables with smoothing parameters (k = number of “knots”) set to 3, in order to allow for a non-linear temperature development through the season and along the CSE whilst avoiding overfitting, whilst *Year* is treated as a categorical factor (i.e. one parameter per year). 1056 temperature profiles from CTD stations and bottle sampling between November and January were downloaded from the ICES hydrographic database [16] and used to fit the model using the “mgcv” package in R [15]. Model building was done by sequentially removing non-significant parameters (*i.e*. those with p>0.05). The final model was then used to predict a time series of temperatures in early winter (15^th^ of December), at the center of the area (1326 km along CSE axis from starting point) where mackerel were known to be present [8].

For validation purposes, we compared the GAM temperature time series with

- a similarly modeled time series further upstream (west of Scotland, 35 km from CSE in the area 55–65°N 10°W-5°E) in February–March, and
- a coarser modeled and validated dataset of sea surface temperatures (SST) obtained from the Hadley Centre SST data set (HadSST2) [17], by averaging over a larger geographical box covering the North Sea-SE Norwegian Sea area (55°N- 65°N, 0–5°E) and including the months from November to January.

Finally, correlation analysis of the mackerel distribution metrics described above and modelled temperature field were performed. All correlation analyses were adjusted for autocorrelation if this exceeded the 95% confidence limits of white noise (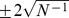, where *N* is sample size) [18]. Adjustments were done by substituting the degrees of freedom with the effective number of degrees of freedom [19].

## Results

**Figure 3:**
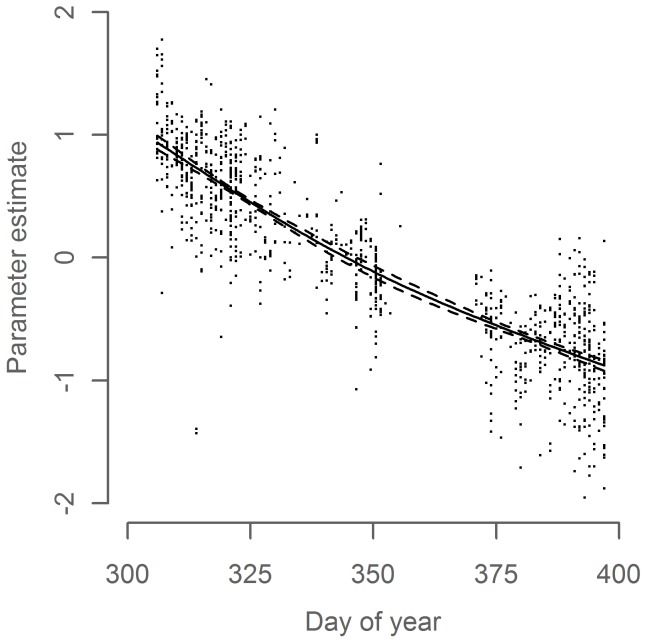
*Day of Year* parameter in the temperature model. Parameter estimate (solid line) with 95% confidence interval (dashed lines) and partial residuals (dots) relative to mean predicted value.

**Figure 4:**
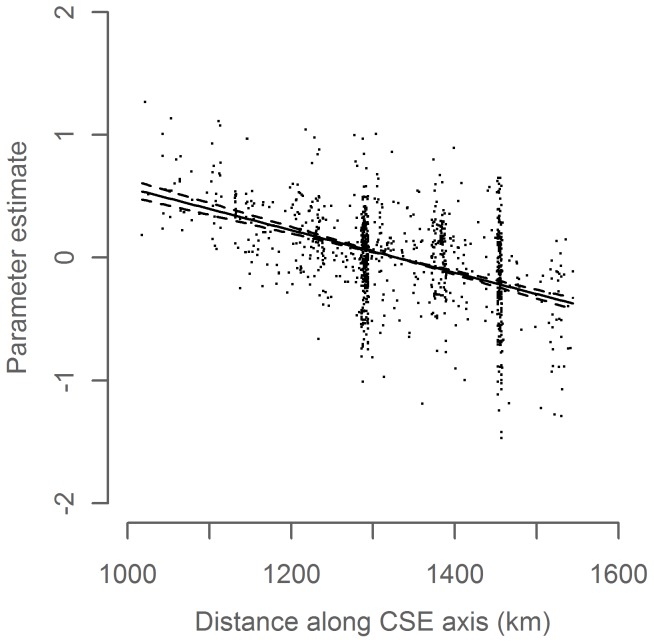
*CSE* parameter in the temperature model. Parameter estimate (solid line) with 95% confidence interval (dashed lines) and partial residuals (dots) relative to mean predicted value.

**Figure 5:**
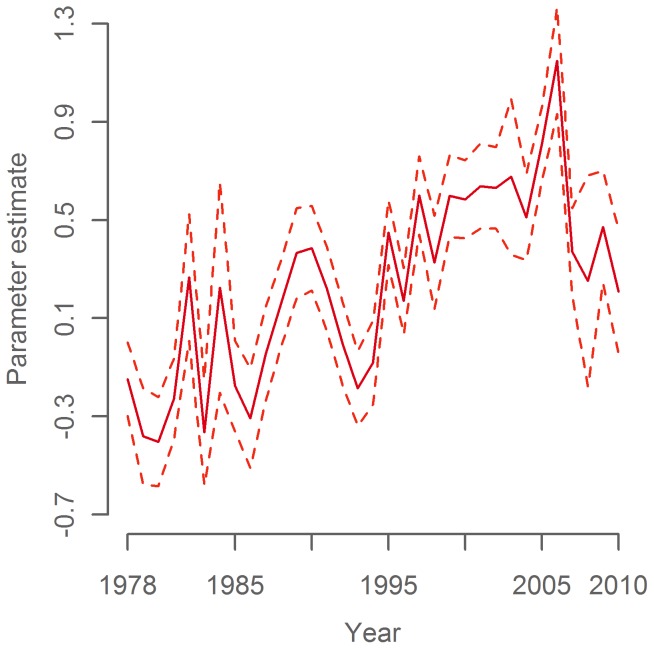
*Year* parameter in the temperature model. Parameter estimate (solid line) with 95% confidence interval (dashed lines) relative to mean predicted value.

The final temperature model identified *Year*, *Day of Year* and *CSE* as significant explanatory variables. In line with expectations, temperature decreased through the winter (Figure 3, p<0.001) and downstream along the CSE axis (Figure 4, p<0.001). The modeled temperature time series shows an overall increase throughout much of the study period with a decrease in the most recent years (Figure 5). The model explained 81% of the variance in the data (adj. R^2^ = 0.81). Parameter estimates for all years are given in table S1. As a rough validation for the overall development of the temperature time series, we found it to be significantly positively correlated to a modeled temperature time series in the area west of Scotland in February–March 1985–2010 (P = 0.005, R^2^ = 0.36, Figure S1, same GAM model structure as the primary temperature series), and also to the Hadley time series of sea surface temperature in November–January 1948–2010 (P<0.001, R^2^ = 0.48, Figure S2).

**Figure 6:**
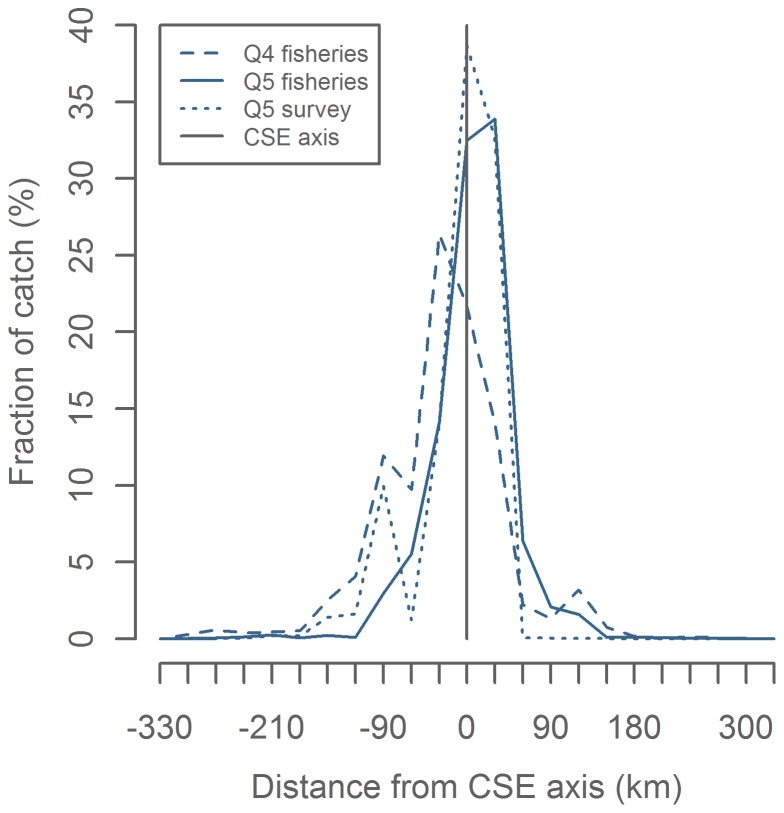
Distance from catch position to continental shelf edge (CSE) axis. Positive values are off the shelf. In the North Sea positive values are northeast of the axis.

**Figure 7:**
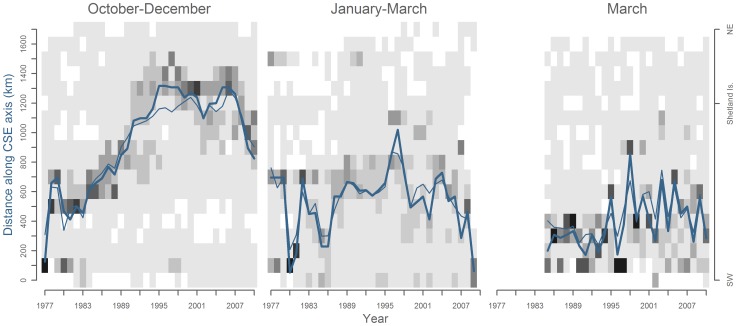
Hovmüller plot of mackerel distributions proxies from commercial landings in October to December (left), January to March (mid) and bottom trawl surveys in March (right). The spatial aspect have been reduced to one dimention by projecting the catch location onto the CSE axis. Greyscale in cells range in 10%-steps from 0–10% (white), to 90–100% (black). Thick line represents position of 50% cumulative landings (left, mid) or CPUE (right) and thin line shows the center of gravity of the distances.

There was a strong tendency for commercial and bottom trawl catches to be associated with the area along the CSE axis, with 74% of the commercial landings in Q4, 92% in Q5 and 87% of the survey catches were taken within a 75 km distance of the CSE axis (Figure 6). We therefore chose to reduce the complexity of the spatial distributions by disregarding the across-axis information, *i.e.* considering the catches projected onto the CSE axis. Visual inspection of Center of Gravity (CoG) and Position of 50% Cumulative Landings (Po50%CL) overlaid on the distributions (Figure 7) indicates that both metrics are appropriate representations of the commercial landings and survey catches.

Landings in Q4 followed a consistent spatial pattern with generally small variance within and between years (Figure 7, left). Landings in Q5 and especially bottom trawl survey catches show greater variance (Figure 7, mid-right).

A progressive southwesterly shift along the CSE axis is evident in the commercial landings data from quarter 4 to 5 (Figure 7, left-mid) and also in the survey catches in late Q5 (Figure 7, right). The average shift of the CoG was found to be 360 km from Q4 to Q5, and 140 km from landings in Q5 to the survey in late Q5.

On a decadal scale, commercial landings (Figure 7, left-mid) show spatial shifts of the commercial fisheries over several hundreds of kilometers, consistent with that reported in the literature [4].

A literature review and an interview with an experienced fishing skipper with first-hand experience of the mackerel fishery during the study period (Tables 1, 2), suggests that factors other than the distribution of mackerel could have influenced the behavior of the fishing fleet, particularly for the Q4 fishery between 1990–1995 and also prior to 2000 for Q5 (see Tables 1, 2). After the collapse of the North Sea Mackerel stock in the 1970s, management measures were put in place in an attempt to protect the remainder of the population [20]. However, since Western and North Sea mackerel mix and are present in the northern North Sea at various times of the year, effective area based management proved difficult. Individual country quotas restricted vessel movements and their ability to target the migrating mackerel. Compounded by the temporal and spatial variability in the migration, this lead to significant misreporting of commercial catch between areas IVa and VIa (and to a lesser extent between IIa and IVa), especially during the 1990s. Incremental changes were made to the management regimes in an attempt to mitigate this misreporting, including partial relaxation of the area-based quotas, modifying area closures, and increased monitoring of the fishery.

**Table 1:** Factors affecting spatiotemporal distribution of the commercial fishery in Q3–4.

Years	Q3–4
1977–1983	Landings data reflected the traditional Q3 Norwegian fishery in the Northern North Sea, and the development of Q3 fisheries more coastal to Eastern Scotland and in the Minches.
1984–1995	The Q3 landings reflect a putative temporal and spatial change in fish availability. Main landings were caught progressively later (ending up in Q4) and north-eastwards from 1983 to 1997 [7]. The large north-eastwards shift from the mid-1980s to mid-1990s occurred in times when fisheries were developing and legislation were changing. However, fisherman observations confirm the spatial development of the fishery was, at least in the beginning, a response to changes mackerel migration patterns as they encountered the mackerel progressively further north-east (Pers. Com. Capt. Alex Wiseman, July 2011). This statement seems reliable, because if the mackerel had been available further north-east in the late 1970s and early 1980s, it would have been economically beneficial to fish on those schools rather than steaming all the way to the Minches from the pelagic ports in north-east Scotland. Later, this fishery (now a Q4 fishery) fluctuates between the coast of Norway and the Shetlands, but remains predominantly east of 4°W.
1996–2010	From about 1996 onwards the fishery was well established in Q4, and its movements through this period was not known to be affected by other large changes than movements of the mackerel stock.

**Table 2:** Factors affecting spatiotemporal distribution of the commercial fishery in Q5.

Years	Q5
1977–1983	Fishery was predominantly in the Cornwall area. However, in this period a new fishery was developing to the north-west of Ireland and west of Scotland
1984	The area around Cornwall was then closed in 1984 to protect the juveniles in this nursery area
1985–1990	The bulk of the landings were from the north of Ireland and west of Scotland moving progressively northwards. The fishery were mainly targeting adult mackerel when they were resident in an area or migrating slowly. However, during this period, development of the pair-trawling technique facilitated the fishery on fast migrating mackerel. Movement of landings in this period may therefore represent a development of the fishery as well as a movement of the stock.
1991–1999	Landings are clustered west of 4 W. This may reflect area misreporting from further east, as the northern North Sea was closed from 31^st^ December.
2000–2010	From 1999 legislation were changed to allow fishing in the northern North Sea up to the 15^th^ of February, and even though this should have ended area misreporting (as the fish were available in the northern North Sea at this time) there appears to have been a “habit” of misreporting to a series of rectangles on the 4 W line which persisted [35].

Further data analysis was restricted to periods where the influence of management measures on the fleet behavior was expected to be minimal. This restricted the landings data from Q5 to only 10 observations (2000–2009), and is therefore why we draw our main conclusions based on the correlation analysis of landings in Q4 and scientific surveys.

**Figure 8:**
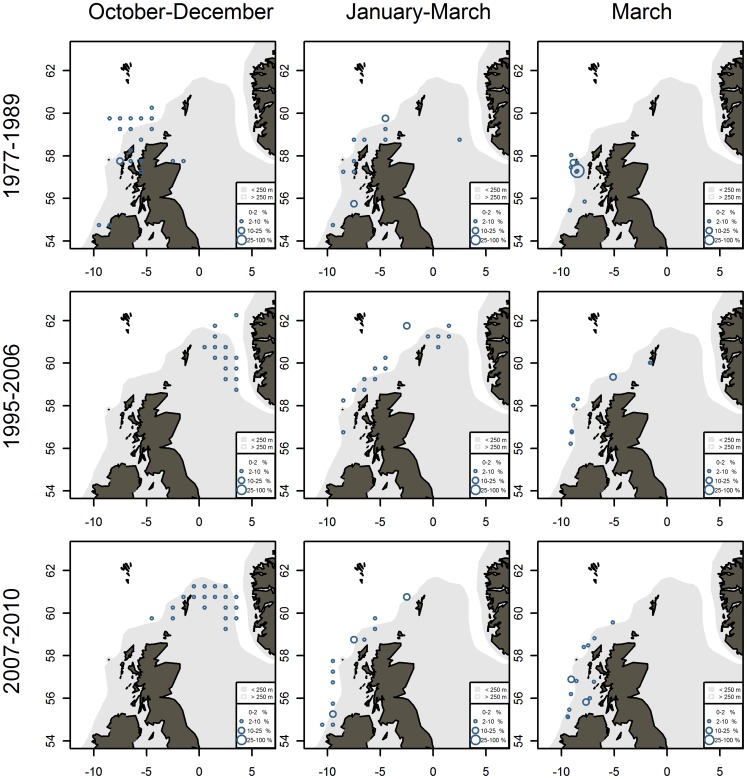
Relative distribution of mackerel landings from the commercial fisheries and mackerel catches from fisheries independent bottom trawl surveys. Data from January–March are shifted back one year to match data in the same season from October–December.

The spatial development of the fishery (Figure 7) during these periods, shows i) a southwestern distribution in Q4 in 1977–1989, ii) a steady northeastern distribution in 2000–2007 (Q4+Q5), followed by iii) a movement toward southwest in 2008–2010 (Q4+Q5). Detailed maps of relative distributions of commercial landings and CPUE from bottom trawl survey in these three periods confirm this pattern **(** **Figure 8** **)**. Annual maps of relative distributions as well as annual and periodic maps of actual catches are given in Figure S3.

An examination of the consistency between the three Po50%CL proxies for spatial distribution showed significant positive correlations between the quarter 4 fisheries and the quarter 5 trawl survey (1985–2010 ex.1990–1995, p = 0.031, R^2^ = 0.23). This was also the case when the quarter 4 and quarter 5 fisheries were analysed (2000–2009, p = 0.040, R^2^ = 0.43). However, no significant correlation was found between the short time series of commercial landings in Q5 and the trawl survey (2000–2009, p>0.05).

**Figure 9:**
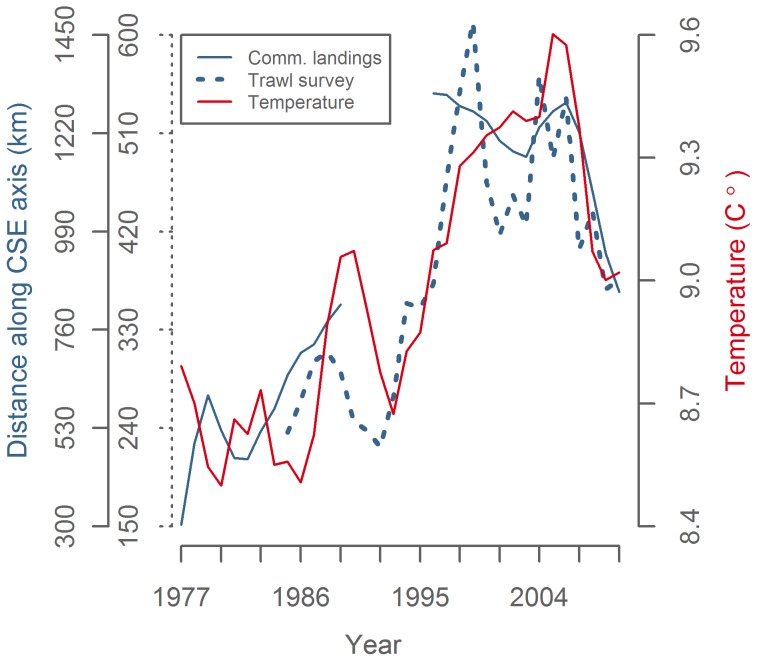
Mackerel distribution and temperature trends (3y rm). Position of 50% cumulative landings in Q4 and in survey CPUE along the continental shelf edge axis (blue) and temperature around the shelf edge in the Northern North Sea from off Shetland Is. to the western edge of the Norwegian trench in November–January (red).

Comparisons of the modelled temperature time series with the Po50%CL proxies for mackerel distribution (Figure 9) reveal a significant positive correlation with fisheries-independent surveys (1985–2010, p = 0.007, R^2^ = 0.27), and with commercial landings in Q4 from 1977–2010 (ex. 1990–1995) (p<0.001, R^2^ = 0.59), but not with the short time series of commercial landings in Q5 (2000–2009, p>0.05). Correlation analyses are summarized in table 3.

**Table 3:** Correlation analyses between proxies for spatial patterns of the mackerel represented by Position of 50% Cumulative Landings (Po50%CL) and modeled temperature in the shelf edge current.

	Landings Q5	Trawl survey	Temperature
**Landings Q4**	p = 0.040, R^2^ = 0.43	p = 0.020, R^2^ = 0.25	p<0.001, R^2^ = 0.58
**Landings Q5**		p>0.05	p>0.05
**Trawl survey**			p = 0.007, R^2^ = 0.27

## Discussion

Our analyses demonstrate that when the NEA mackerel return in late summer from the feeding areas on the European shelf and in the Nordic Seas [4], they aggregate through autumn and early winter along the continental shelf edge, where they are targeted by commercial trawlers and purse seiners. Later in winter the commercial fleets and the fisheries independent bottom trawl survey find the mackerel further towards the southwest. The path of the migration, as suggested by the location of commercial and survey catches coincides with the location of the relatively warm high-saline eastern Atlantic water flowing north-eastwards on and along the continental shelf edge, flanked by cooler water masses. We present a modelled new time series of temperature in this current and find it to be significantly correlated with two proxies for spatiotemporal mackerel distribution. The proxies are derived from data over a significant period of time and a large proportion of the European shelf and encapsulate large scale changes in distribution. Our results indicate that

- the mackerel population is found further upstream in warmer waters as the current cools through winter
- this process is associated via climatic variability, with large impacts on the mackerel migration and fisheries, and suggest a mechanism where
- this affinity for warm water leads the mackerel towards the main spawning areas.

These results are in accordance with earlier studies of mackerel during autumn and winter [5]–[10].

The present work illustrates the limitations associated with the available data and underlines that caution should be exercised when utilising catch data as a proxy for distribution. The relatively low trawling speed and small scale trawls employed by standardized scientific surveys are unsuited for catching a fast pelagic species like mackerel. Furthermore, changes in vertical distribution and schooling behaviour reduce the signal-to-noise ratio in the trawl survey data and contributes to the low levels of explained variance (R^2^) in correlations that include this variable. In contrast, commercial fishing employs much more efficient methods. Commercial landings data are, however, only appropriate for inferring changes in stock movements over time when other factors remain relatively constant. This was not the case for the Q4 fishery between 1990 to 1995, when the management regime restricted the ability of vessels to target fish migrating through areas IVa and VIa and fisheries technology and techniques changed the behaviour and increased the efficiency of the fleet (Table 1). An approach to circumvent this problem has been used in a previous study, where high resolution catch data from a validated subset of the fleet showed that the observed change from late 1970s to late 1990s leveled out from 1989 to 1994 [5]. This is consistent with our conclusions, as this was the period where fisheries and temperature deviated (Figure 9).

Other major changes in mackerel fisheries have occurred through the period 1977–2010, such as the summer fishery in Icelandic waters that commenced in recent years [4]. While this fishery is outside the main scope of this study, it is related to the westward expansion of the summer distribution [21]. Changes in the summer distribution could lead to a change in the path taken during the return migration in late summer and early autumn, which could potentially affect the autumn-winter distribution. Further investigation of this effect is therefore warranted.

The results presented are in accord with recent investigations that link climatic variability and spatiotemporal dynamics of mackerel spawning [12], [22], [23], [33]. Mackerel differ from most other exothermal organisms by being i) purely pelagic through all life stages, and ii) relatively fast and constantly swimming [24], able to react to the environment by migrating over long distances. This dynamic spatial behavior enables the mackerel to avoid poor temperature conditions during its migration in search of optimal areas for reproduction and feeding. This seems to be most evident during the cold season when other constraints such as feeding and reproduction are reduced or absent. The effect of temperature on the spatial shifts of the mackerel distribution is suggested to be on a scale of hundreds of kilometers during winter (Figure 9), much larger than in spring where spawning has been moving only 40 km north per °C [23] and in summer where polar water merely forms an outer boundary of the extremely large area occupied by mackerel [4], [25]. It is understood that the primary activity during winter is the maturation of eggs and sperm. It may be that the specific temperature conditions selected by the mackerel are an adaptation to optimize development of reproductive products. The present findings facilitate testing of this hypothesis and exploration of further importance for spawning.

The physical environment within the shelf edge current is related to large scale oceanographic circulation patterns. Conditions in the Bay of Biscay and the European shelf seas, to the east of the continental shelf edge current, are related to the Northern Hemisphere Temperature trend [26]. This differs from the oceanic region west of the shelf edge current, which to a greater extent is regulated by the dynamics of the subpolar gyre [27], [28]. The physical environment within the shelf edge current is related to the northern hemisphere temperature type of variability, but may also be influenced by the oceanic domain during periods when the subpolar gyre circulation is particularly strong, such as during the period 1990–1995 [27]. The shelf edge waters are furthermore modulated by smaller sub-decadal oscillations, caused by pulses of eastern water from the Bay of Biscay [29]. Once warm and saline anomalies have passed the Porcupine Bank, the geographic divide between the subtropical and the subpolar gyres, they are destined to continue northward as baroclinic Rossby waves [30], [31], with an advection time of one-two years, to the entrance of the Nordic Seas [27], [32]. This oceanic inertia holds promise for making projections one-two years into the future. Shorter-term predictions may be possible based on measurements of the temperature further “upstream”: such predictions could be of value for the fishing industry as it may reduce the time spend on searching for mackerel. However, detailed forecasting of mackerel behavior outside the observed temperature range is not possible before any additional causal effects and their interactions are sufficiently clarified.

The results presented have implications for the management, fishery and monitoring of mackerel. Recent changes in mackerel distribution have resulted in political disputes over zonal attachments and led to a break-down of the international management agreements since 2008. Furthermore, in 2009 fishermen were taken by surprise when the mackerel had departed the northern North Sea east of 4° (which separates management areas IVa and VIa) by October [34], significantly earlier than in previous years. As a consequence, quotas worth over 100 M € could not be utilized in that year by the Norwegian and Danish industries [35] whilst, at the same time, Scottish seiners had little difficulty in catching the mackerel further west. We have demonstrated that cooling of the continental shelf edge current, possibly triggered this early migration. In a climate change scenario where temperatures increase further, our results suggest that mackerel distribution is likely to be affected with subsequent effects for the fishery and mackerel prey.

## Supporting Information

Figure S1 **Temperature time series from November–January 1977–2010 northern North Sea used in the analysis of mackerel distributions (solid line as 3 year running mean).** Temperature time series from February–March 1985–2010 west of Scotland (dashed line as 3 year running mean). Both series modeled as described in material and methods.
(TIF)

Figure S2 **3 year running means of temperature time series 1948–2010.** Red: Primary temperature series in November–January northern North Sea. Modeled as described in material and methods for the shorter time series. Black: Hadley sea surface temperature anomaly in November–January 55–65 N 10 W–5 E (black). Data from Hadley Centre SST data set (HadSST2) [17].
(TIF)

Figure S3 **Mackerel landings from commercial fisheries and mackerel catches from fisheries independent bottom trawl surveys.** Data from January–March are shifted back one year to match data in the same season from October–December.
(TIF)

Table S1 Table with temperature model parameter estimates.
(DOC)
