# Supplementary figures and images for: Migration and Fisheries of North East Atlantic Mackerel (Scomber scombrus) in Autumn and Winter
Source: PLoS One. 2012 Dec 10;7(12):e51541. doi: 10.1371/journal.pone.0051541 (PMC3519697; doi:10.1371/journal.pone.0051541)

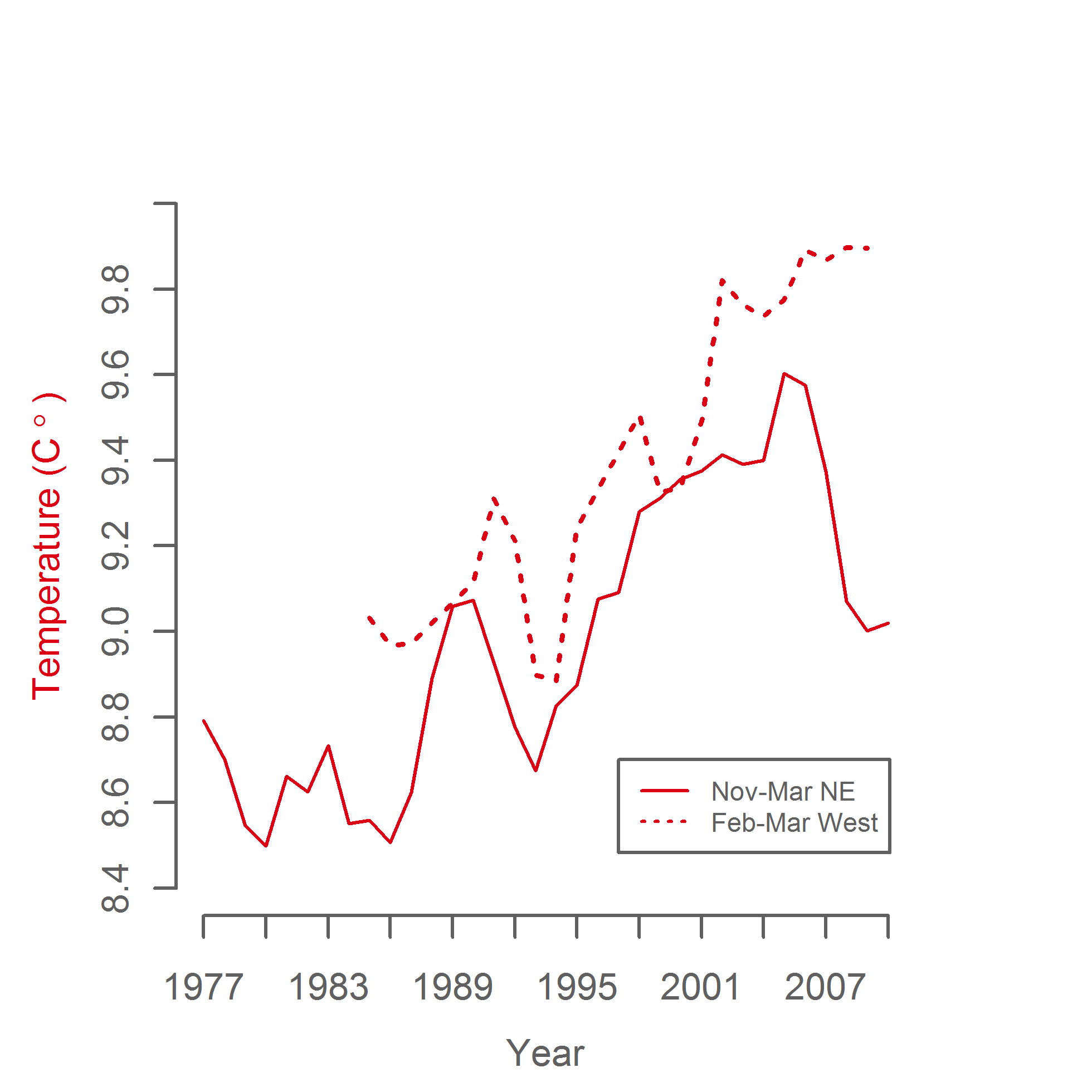

Supplement: Figure S1 — Temperature time series from November–January 1977–2010 northern North Sea used in the analysis of mackerel distributions (solid line as 3 year running mean). Temperature time series from February–March 1985–2010 west of Scotland (dashed line as 3 year running mean). Both series modeled as described in material and methods. (TIF) [file pone.0051541.s001.tif]

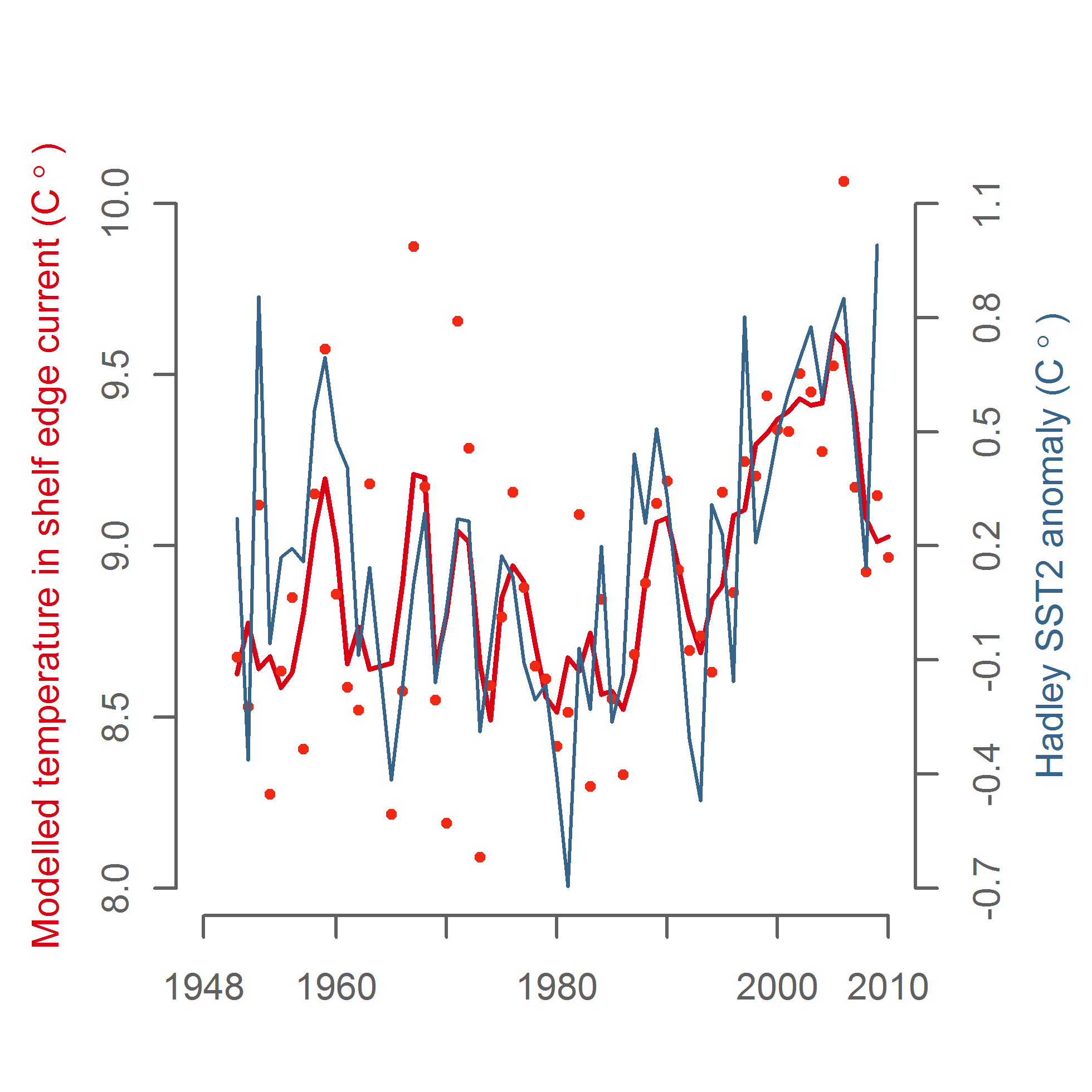

Supplement: Figure S2 — 3 year running means of temperature time series 1948–2010. Red: Primary temperature series in November–January northern North Sea. Modeled as described in material and methods for the shorter time series. Black: Hadley sea surface temperature anomaly in November–January 55–65 N 10 W–5 E (black). Data from Hadley Centre SST data set (HadSST2) [17]. (TIF) [file pone.0051541.s002.tif]

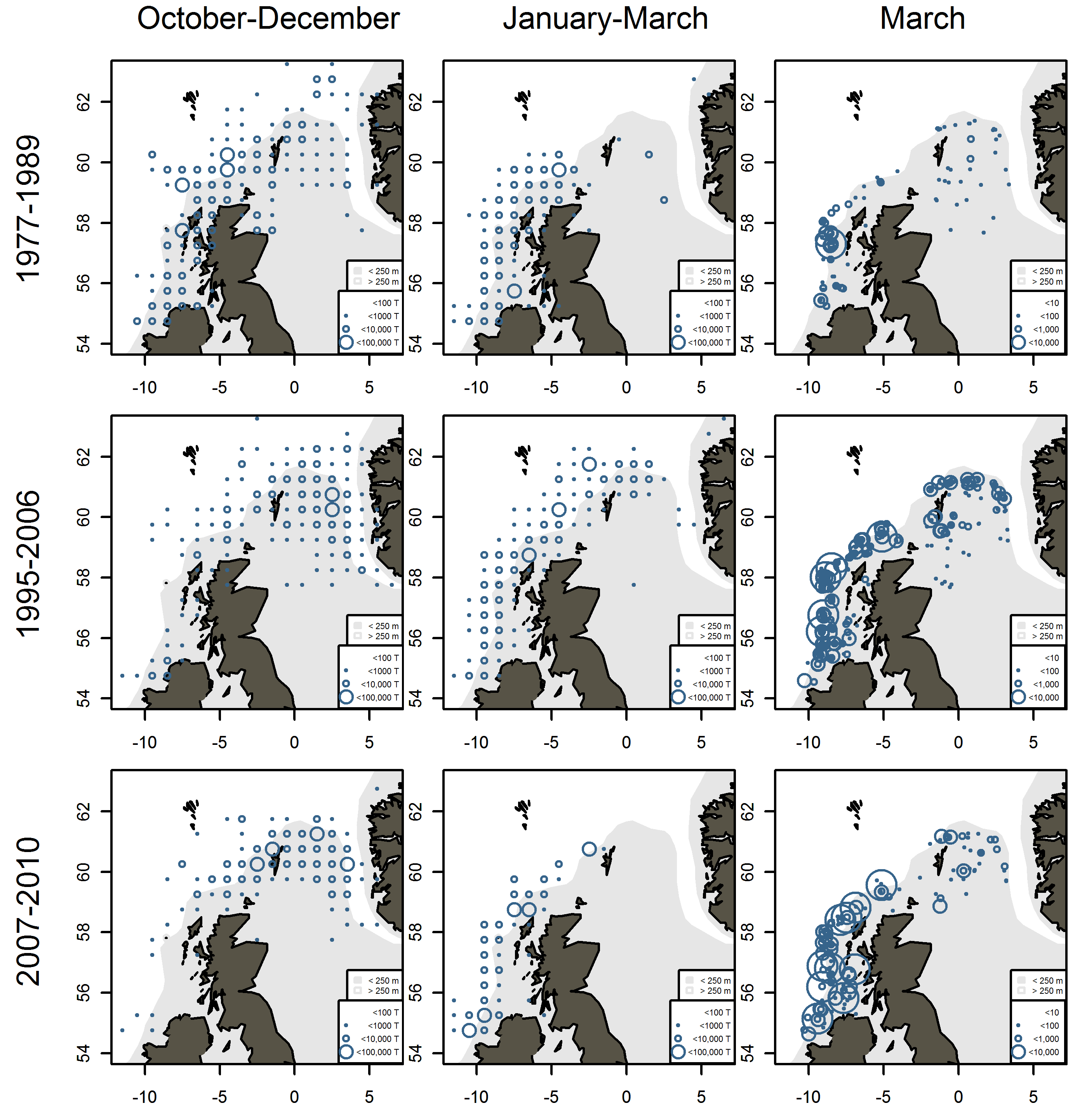

Supplement: Figure S3 — Mackerel landings from commercial fisheries and mackerel catches from fisheries independent bottom trawl surveys. Data from January–March are shifted back one year to match data in the same season from October–December. (TIF) [file pone.0051541.s003.tif]
