# Supplementary material for: Migration and Fisheries of North East Atlantic Mackerel (Scomber scombrus) in Autumn and Winter
Source: PLoS One. 2012 Dec 10;7(12):e51541. doi: 10.1371/journal.pone.0051541 (PMC3519697; doi:10.1371/journal.pone.0051541)
Supplement: Table S1 — Table with temperature model parameter estimates. (DOC) [file pone.0051541.s004.doc]

| **Parameter** | **Estimate** | **St. dev.** | **p-value** |
| --- | --- | --- | --- |
| **Intercept** | 8.71 | 0.06 | < 0.001 |
| ***Year 1978*** | -0.15 | 0.08 | 0.048 |
| ***Year 1979*** | -0.38 | 0.10 | < 0.001 |
| ***Year 1980*** | -0.40 | 0.10 | < 0.001 |
| ***Year 1981*** | -0.23 | 0.08 | 0.006 |
| ***Year 1982*** | 0.27 | 0.13 | 0.0396 |
| ***Year 1983*** | -0.36 | 0.11 | < 0.001 |
| ***Year 1984*** | 0.22 | 0.21 | 0.296 |
| ***Year 1985*** | -0.18 | 0.09 | 0.056 |
| ***Year 1986*** | -0.31 | 0.10 | 0.003 |
| ***Year 1987*** | -0.04 | 0.10 | 0.638 |
| ***Year 1988*** | 0.16 | 0.09 | 0.073 |
| ***Year 1989*** | 0.37 | 0.09 | < 0.001 |
| ***Year 1990*** | 0.39 | 0.09 | < 0.001 |
| ***Year 1991*** | 0.22 | 0.09 | 0.011 |
| ***Year 1992*** | < -0.01 | 0.08 | 0.963 |
| ***Year 1993*** | -0.19 | 0.08 | 0.015 |
| ***Year 1994*** | -0.08 | 0.09 | 0.343 |
| ***Year 1995*** | 0.45 | 0.07 | < 0.001 |
| ***Year 1996*** | 0.17 | 0.07 | 0.011 |
| ***Year 1997*** | 0.60 | 0.08 | < 0.001 |
| ***Year 1998*** | 0.33 | 0.10 | < 0.001 |
| ***Year 1999*** | 0.60 | 0.08 | < 0.001 |
| ***Year 2000*** | 0.58 | 0.08 | < 0.001 |
| ***Year 2001*** | 0.64 | 0.09 | < 0.001 |
| ***Year 2002*** | 0.63 | 0.08 | < 0.001 |
| ***Year 2003*** | 0.68 | 0.16 | < 0.001 |
| ***Year 2004*** | 0.51 | 0.09 | < 0.001 |
| ***Year 2005*** | 0.81 | 0.08 | < 0.001 |
| ***Year 2006*** | 1.15 | 0.11 | < 0.001 |
| ***Year 2007*** | 0.37 | 0.09 | < 0.001 |
| ***Year 2008*** | 0.25 | 0.21 | 0.240 |
| ***Year 2009*** | 0.47 | 0.11 | < 0.001 |
| ***Year 2010*** | 0.21 | 0.13 | 0.102 |
| ***Day of Year*** | NA | NA | < 0.001 |
| ***CSE*** | NA | NA | < 0.001 |
